# Association between preoperative systemic immune inflammation index and postoperative sepsis in patients with intestinal obstruction: A retrospective observational cohort study

**DOI:** 10.1002/iid3.1187

**Published:** 2024-02-14

**Authors:** Jirong Yang, Taojia Ran, Xiaoyu Lin, Jinyan Xu, Shaoli Zhou, Chaojin Chen, Pinjie Huang

**Affiliations:** ^1^ Department of Anesthesiology The Third Affiliated Hospital of Sun Yat‐sen University Guangzhou People's Republic of China

**Keywords:** intestinal obstruction, risk factor, sepsis, systemic immune inflammation index

## Abstract

**Background:**

Sepsis is a severe complication that results in increased morbidity and mortality after intestinal obstruction surgery. This study examined the role of preoperative systemic immune inflammation index (SII) for postoperative sepsis in intestinal obstruction patients.

**Methods:**

Data on patients who underwent intestinal obstruction surgery were collected. SII was determined and separated into two groups (≤1792.19 and >1792.19) according to the optimal cut‐off value of SII for postoperative sepsis. The odds ratio (OR) is calculated for the correlation between SII and postoperative sepsis. Additional analyses were used to estimate the robustness of SII.

**Results:**

A total of 371 intestinal obstruction patients undergoing surgery were included in the final cohort, and 60 (16.17%) patients developed postoperative sepsis. Patients with an SII ＞1792.19 had a significantly higher risk for developing postoperative sepsis after multivariable adjustment [adjusted odds ratio = 2.12, 95% confidence interval: [1.02–4.40]]. The analysis of interaction showed no correlation between the preoperative SII and postoperative sepsis regarding age, hypertension, American Society of Anesthesiologists classification, blood loss, albumin, hemoglobin, creatinine, and leukocyte (all interactions *p* > .05). In subgroup analysis, all statistically significant subgroups showed that SII was a risk factor for postoperative sepsis (all *p* < .05). The analyses of subgroups and interactions revealed that the interaction effect of a preoperative SII ＞1792.19 and postoperative sepsis remained significant. A sensitivity analysis confirmed the robustness of the results.

**Conclusions:**

A preoperative SII ＞ 1792.19 was a risk factor for postoperative sepsis in patients undergoing intestinal obstruction surgery.

## Graphical abstract

The systemic immune inflammation index (SII), derived from laboratory testing, serves as a risk factor for postoperative sepsis in patients with intestinal obstruction. With the increase of SII, the risk of postoperative sepsis is higher.

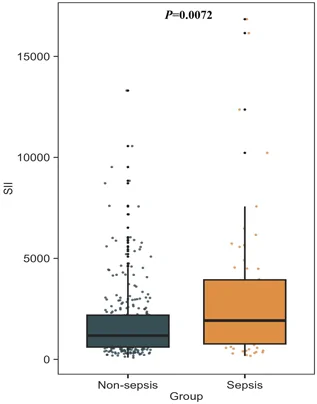

## 1 INTRODUCTION

Sepsis is described as life‐threatening organ dysfunction resulting from a maladjusted response to infection^1^ and is a syndrome of physiological, morphological, and metabolic dysfunction caused by infection. Sepsis has a rising documented incidence. Although the actual incidence is unclear, sepsis is the leading cause of global death and critical illness according to a conservative estimate.^2^, ^3^ The International Guidelines for Management of Sepsis and Septic Shock^4^ recommended starting antibacterial treatment within 1 h after the onset of sepsis. However, due to the complexity of the clinical environment^5^ and the different clinical presentations of sepsis according to its cause and population,^6^ it is challenging to accurately identify sepsis early, while delayed treatment often leads to poor prognosis.^7^, ^8^ In addition to being the organ in charge of nutritional absorption, the gastrointestinal tract also has immune and metabolic functions and serves as a barrier against bacteria and endotoxins in the intestinal lumen.^9^ Intestinal obstruction surgery is considered a high‐risk procedure for sepsis.^10^ Although without intestinal perforation and necrosis, patients with intestinal obstruction rarely experience sepsis before surgery. However, intestinal obstruction patients are prone to malnutrition, bacterial translocation,^11^, ^12^ changes in bacterial diversity of the obstructed intestinal segments,^13^ surgical trauma and the effects of anesthesia on patients, as well as a series of pathophysiological changes caused by complex perioperative factors; as such, sepsis can easily occur after surgery.

In recent years, due to their low cost and straightforward availability, an everbroader range of biological markers have been applied in clinical practice. Several studies have found some inflammatory biomarkers to be associated with the diagnosis and prognosis of sepsis, including procalcitonin (PCT), C‐reactive protein‐to‐albumin ratio (CAR), and neutrophil‐to‐lymphocyte ratio (NLR).^14^, ^15^, ^16^ However, these predictors tended to become unstable and vulnerable to the effect of additional confounding variables when just one or two characteristics were involved.^17^ The systemic immune inflammation index (SII), obtained by dividing the product of neutrophils and platelets by lymphocyte count (LYMPH), is a novel biomarker associated with outcomes of bladder cancer,^18^ gastroesophageal adenocarcinoma,^19^ and colorectal cancer.^20^ There is also some connection between sepsis and SII. It has been reported that SII can be used in addition to clinical sepsis scores to enhance the accuracy of patient evaluation.^21^ Meanwhile, a retrospective study showed that SII was associated with short‐term mortality in the population of critically ill patients with sepsis.^22^ However, there have been limited studies on the inflammatory factors in patients with intestinal obstruction who are at a high risk of developing postoperative sepsis. We speculated that SII is a risk factor for sepsis after intestinal obstruction surgery. This retrospective study explored the relationship between preoperative SII and postoperative sepsis in patients with intestinal obstruction.

## 2 METHODS

### 2.1 Data sources and patients

This is a single‐center retrospective observational cohort study. Patients with intestinal obstruction who underwent surgery from April 2013 to April 2021 at the Third Affiliated Hospital of Sun Yat‐sen University were included in the cohort. Patients who meet the following exclusion criteria were omitted from this study: minors, pregnant women, patients who underwent secondary procedures for intestinal obstruction, and patients who lacked sufficient relevant records. Ethical approval of this study was obtained from the Research Ethics Committee at the Third Affiliated Hospital of Sun Yat‐sen University (NO. [2022] 02‐004‐02). This study was exempted from having to obtain informed consent because the patient identities would not be recognized, and perioperative data were obtained from electrical health records (EHR). All data were collected and verified by three physicians to eliminate potential biases. They independently extracted the data according to the unified inclusion and exclusion standard, and then jointly checked the data to ensure accuracy. All inflammatory indicators were taken from the latest preoperative blood routine test. This report adheres to the strengthening the reporting of observational studies in epidemiology checklist for observational studies.

### 2.2 Data collection

Based on clinical experience and existing studies, clinical data including demographics, clinical characteristics, laboratory values, or perioperative variables associated with sepsis and intestinal obstruction were derived from EHRs. The demographics and preoperative comorbidities included age, gender, hypertension, coronary disease, diabetes, respiratory disease, American Society of Anesthesiologists (ASA) classification, New York Heart Association classification, preoperative shock, and preoperative organ failure. Characteristics of the disease including obstruction site, cause, nature, degree, and intestinal state. Baseline laboratory findings included white blood cell (WBC) count, hemoglobin (HGB), neutrophil count (NEUT), LYMPH, monocyte count (MONO), platelet count (PLT), albumin (ALB), and creatinine (CR). Intraoperative variables included blood loss, urine volume, operation duration, and anesthesia duration.

### 2.3 Outcomes and exposures

The main outcome was postoperative sepsis diagnosed according to the Sepsis 3.0 diagnostic criteria.^1^ Sepsis can be diagnosed if the patient's sequential organ failure assessment (SOFA) score is greater than or equal to 2, based on the infection or suspected infection. The SOFA score is mainly obtained through a comprehensive evaluation of circulation, PLT, TBIL, GCS, Cr, and PaO_2_/FiO_2_. The incidence of sepsis during postoperative hospitalization was recorded. Based on the sepsis criteria, patients were separated into two groups, that is, a sepsis group and a nonsepsis group. The main exposure was computed as the quotient of neutrophils and platelets divided by LYMPHs, that is, the SII. All diagnostic data were derived from EHRs. According to the optimal cut‐off value of SII for postoperative sepsis, patients with higher SII were divided into a higher level of SII group, while those with lower SII were divide into a lower level of SII group.

### 2.4 Statistical analysis

The SPSS Statistics (v.22), MedCalc (v.20.022), PASS software (version 15.0 NCSS), and EmpowerStats (http://www.empowerststs.com, X&Y Solutions, Inc.) software packages were used to complete the statistical analysis. The median (interquartile range) was used to represent quantitative variables, while number (%) was employed to express qualitative variables. The quantitative data were examined using the student's *t* test or the Wilcoxon rank‐sum (Mann–Whitney) test, and the qualitative data was evaluated using Pearson's *χ* ^2^ test. A *p* < .05 was considered statistically significant. Missing continuous and categorical variables were filled by means and modes, respectively. The Youden index were obtained from the receiver operating characteristic (ROC) curve analysis. And the Youden's index with the greatest sensitivity and specificity was used to calculate the optimal cut‐off value of the SII for sepsis and to categorize patients into high or low SII groups. The PASS software (version 15.0 NCSS) was used for post hoc power analysis (two‐sided, *Z* test, and *⍺* = .05). The relationship between the preoperative SII and postoperative sepsis in patients with intestinal obstruction was estimated using binary logistic regression. The adjusted correlations between the SII and postoperative sepsis were then investigated across all groups using multivariate logistic regression, which included the predefined confounders of age, duration of operation, WBC, obstruction cause, and intestinal state. Based on the literature and our clinical experience, interaction, and stratified analyses were performed to assess whether the association between postoperative sepsis and the SII had been influenced by confounding factors. To investigate the robustness of this association between the SII and postoperative sepsis, several sensitivity analyses were conducted. We investigated whether the association would change if patients who had experienced shock or organ dysfunction before surgery were excluded. Given the potential impact of preoperative intestinal perforation on postoperative sepsis, we investigated the relationship between the SII and all patients except for preoperative intestinal perforation. For exploratory purposes, we evaluated the relationship between sepsis and postoperative outcomes such as in‐hospital mortality, duration of stay, and total cost.

## 3 RESULTS

### 3.1 Study cohort and characteristics

**Figure 1:**
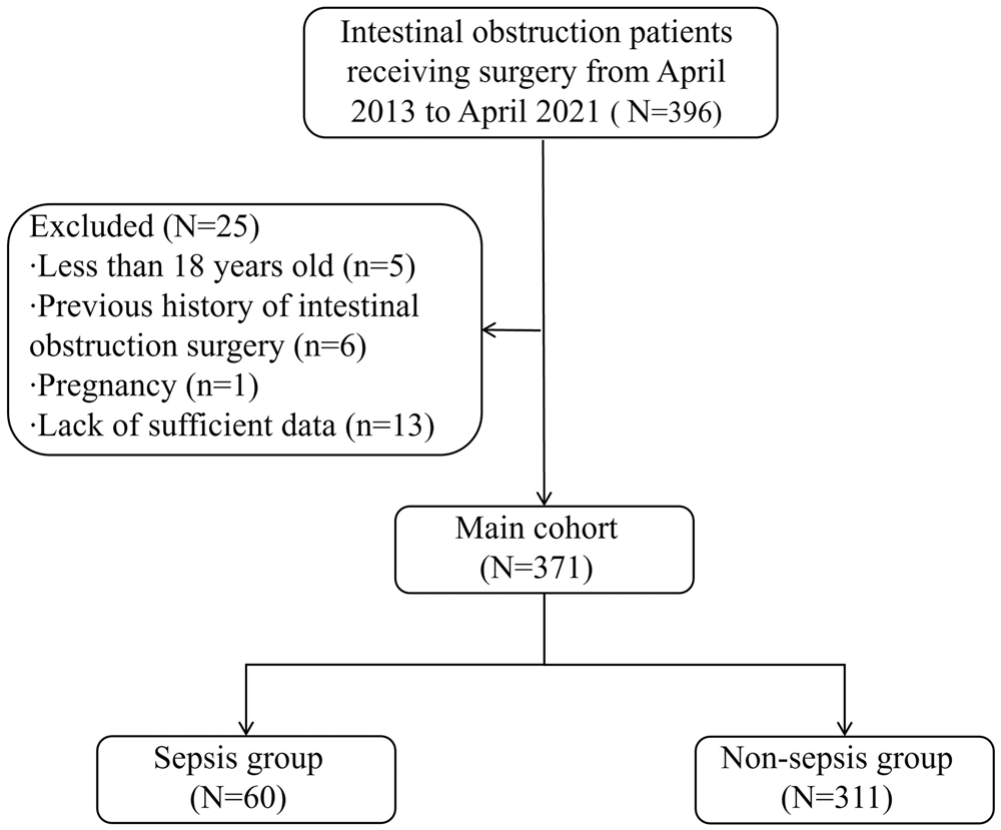
Flow chart of patient enrollment.

The eligibility review comprised 396 individuals receiving surgery for intestinal obstruction. After excluding ineligible patients, the final cohort of 371 individuals included 242 (65.23%) men and 129 (34.77%) women (Figure 1). The median age was 47 years old. Among these patients with intestinal obstruction, 60 (16.17%) patients developed sepsis during hospitalization after surgery (Table 1). Patients aged ≥65 years old had a higher incidence of sepsis (63.33% vs. 33.44%, *p* < .001). Patients with postoperative sepsis were more likely to have hypertension (33.33% vs. 20.26%, *p* = .026), respiratory disease (40% vs. 17.68%, *p* < .001), an ASA classification of III/IV (83.33% vs. 32.48%, *p* < .001), higher WBC (10.17 × 10^−9^/L vs. 8.04 × 10^−9^/L, *p* = .008), higher NEUT (6.12 × 10^−9^/L vs. 5.42 × 10^−9^/L, *p* = .035) and lower LYMPH (0.93 × 10^−9^/L vs. 1.20 × 10^−9^/L, *p* < .001). Patients in the sepsis group experienced longer operative time (218 vs. 188 min, *p* = .004), longer anesthesia time (276 vs. 255 min, *p* = .049), and more blood loss (100 vs. 50 mL, *p* < .001) during surgery. The postoperative prognosis is shown in Table 2. Compared with patients that did not develop sepsis, those who did had a longer time to first postoperative defecation, an extended time of drainage tube retention, a higher total hospitalization cost, longer total hospitalization days, longer postoperative hospitalization days, longer intensive care unit (ICU) length of stay and a higher risk of in‐hospital mortality (all *p* < .001).

**Table 1:** Baseline characteristics between patients with and without sepsis.

Characteristics	Total (*n* = 371)	Sepsis		*p* Value
		No (*n* = 311)	Yes (*n* = 60)	
Age (years)^a^				<.001
≥65	142 (38.27%)	104 (33.44%)	38 (63.33%)	
<65	229 (61.73%)	207 (66.56%)	22 (36.67%)	
Gender^a^				.253
Men	242 (65.23%)	199 (63.99%)	43 (71.67%)	
Women	129 (34.77%)	112 (36.01%)	17 (28.33%)	
Hypertension^a^				.026
Yes	83 (22.37%)	63 (20.26%)	20 (33.33%)	
No	288 (77.63%)	248 (79.74%)	40 (66.67%)	
Coronary disease^a^				.953
Yes	18 (4.85%)	15 (4.82%)	3 (5%)	
No	353 (95.15%)	296 (95.18%)	57 (95%)	
Diabetes^a^				.240
Yes	45 (12.13%)	35 (11.25%)	10 (16.67%)	
No	326 (87.87%)	276 (88.75%)	50 (83.33%)	
Respiratory disease^a^				<.001
Yes	79 (21.29%)	55 (17.68%)	24 (40%)	
No	292 (78.71%)	256 (82.32%)	36 (60%)	
ASA classification^a^				<.001
ASA I–II	220 (59.30%)	210 (67.52%)	10 (16.67%)	
ASA III–IV	151 (40.71%)	101 (32.48%)	50 (83.33%)	
NYHA classification^a^				.043
NYHA I–II	357 (96.23%)	302 (97.11%)	55 (91.67%)	
NYHA III–VI	14 (3.77%)	9 (2.89%)	5 (8.33%)	
Shock^a^				<.001
Yes	16 (4.31%)	2 (0.64%)	14 (23.33%)	
No	355 (95.69%)	309 (99.36%)	46 (76.67%)	
Organ failure^a^				<.001
Yes	19 (5.12%)	7 (2.25%)	12 (20%)	
No	352 (94.88%)	304 (97.75%)	48 (80%)	
Obstruction site^a^				.116
Colorectum	189 (50.94%)	164 (52.73%)	25 (41.67%)	
Small intestine	182 (49.06%)	147 (47.27%)	35 (58.33%)	
Obstruction degree^a^				.072
Completeness	292 (78.71%)	250 (80.39%)	42 (70%)	
Noncompleteness	79 (21.29%)	61 (19.61%)	18 (30%)	
Obstruction cause^a^				.002
Mechanical	354 (95.42%)	302 (97.106%)	52 (86.667%)	
Dynamic	17 (4.58%)	9 (2.894%)	8 (13.333%)	
Obstructive nature^a^				.438
Tumorous	155 (41.78%)	131 (42.12%)	24 (40%)	
Nontumorous	216 (58.22%)	180 (57.88%)	36 (60%)	
Intestinal state^a^				<.001
Normal	275 (74.12%)	247 (79.421%)	28 (46.667%)	
Hyperemia/ischemia	34 (9.16%)	28 (9.003%)	6 (10.0%)	
Necrosis	31 (8.36%)	18 (5.788%)	13 (21.667%)	
Perforation	31 (8.36%)	18 (5.788%)	13 (21.667%)	
Operative time (min)^b^	189 (131, 263)	188 (130, 255)	218 (150, 328)	.004
Anesthesia time (min)^b^	255 (193, 340)	255 (190, 330)	276 (205, 387)	.049
Urine volume (mL)^b^	500 (300, 750)	500 (300, 750)	550 (300, 800)	.516
Blood loss (mL)^b^	50 (50, 100)	50 (30, 100)	100 (50, 200)	<.001
WBC (10^−9^/L)^b^	8.43 (6.15, 12.11)	8.04 (5.91, 11.79)	10.17 (6.59, 14.84)	.008
NEUT (10^−9^/L)^b^	5.51 (3.51, 8.76)	5.42 (3.38, 8.59)	6.12 (4.42, 10.73)	.035
LYMPH (10^−9^/L)^b^	1.17 (0.845, 1.51)	1.20 (0.89, 1.56)	0.93 (0.57, 1.30)	<.001
MONO (10^−9^/L)^b^	0.61 (0.41, 0.83)	0.60 (0.41, 0.83)	0.65 (0.43, 0.86)	.460
PLT (10^−9^/L)^a^				.001
≥100	361 (97.30%)	307 (98.71%)	54 (90%)	
<100	10 (2.70%)	4 (1.29%)	6 (10%)	
ALB (g/L)^a^				.001
≥35	219 (59.03%)	195 (62.70%)	24 (40%)	
<35	152 (40.97%)	116 (37.30%)	36 (60%)	
HGB (g/L)^a^				.016
≥115	213 (57.41%)	187 (60.13%)	26 (43.33%)	
<115	158 (42.59%)	124 (39.87%)	34 (56.67%)	
CR (umol/L)^a^				<.001
>116	34 (9.16%)	19 (6.11%)	15 (25%)	
≤116	337 (90.84%)	292 (93.89%)	45 (75%)	
SII^a^				<.001
>1792.19	130 (35.04%)	96 (30.87%)	34 (56.67%)	
≤1792.19	241 (64.96%)	215 (69.13%)	26 (43.33%)	

Abbreviations: ALB, albumin; ASA, American Society of Anesthesiologists; CR, creatinine; HGB, hemoglobin; LYMPH, lymphocyte count; MONO, monocyte count; NEUT, neutrophil count; NYHA, New York Heart Association; PLT, platelet count; SII, systemic immune inflammation index; WBC, white blood cell.

^a^ Categorical variables are expressed as a number (%).

^b^ Continuous variables are expressed as a median (interquartile range).

**Table 2:** Comparison of postoperative features of the study patients.

Variables	Nonsepsis (311)	Sepsis (60)	*p* Value
ICU length of stay (h)	0.0 (0.0, 0.0)	60.5 (17.3, 124.8)	<.001
Proportion of ICU stays (%)	43 (13.8%)	49 (81.7%)	<.001
Total hospitalization days (d)	17.0 (11.0, 23.0)	21.0 (14.0, 35.0)	<.001
Total hospitalization cost (yuan)	64,817.0 (42,266.0, 83,647.6)	115,072.25 (85,466.7, 162,846.6)	<.001
Postoperative hospitalization days (d)	9.0 (7.0, 12.0)	18.5 (12.0, 29.0)	<.001
Time of first postoperative defecation (day)	3 (3, 5)	4 (4, 6)	<.001
Time of drainage tube retention (day)	4 (2, 6)	7 (5, 12)	<.001
In‐hospital mortality (%)	1 (0.3%)	15 (25%)	<.001

*Note*: Continuous variables are expressed as the median (interquartile range); categorical variables are expressed as a number (%).

Abbreviation: ICU, intensive care unit.

### 3.2 Relationship between SII and sepsis

**Figure 2:**
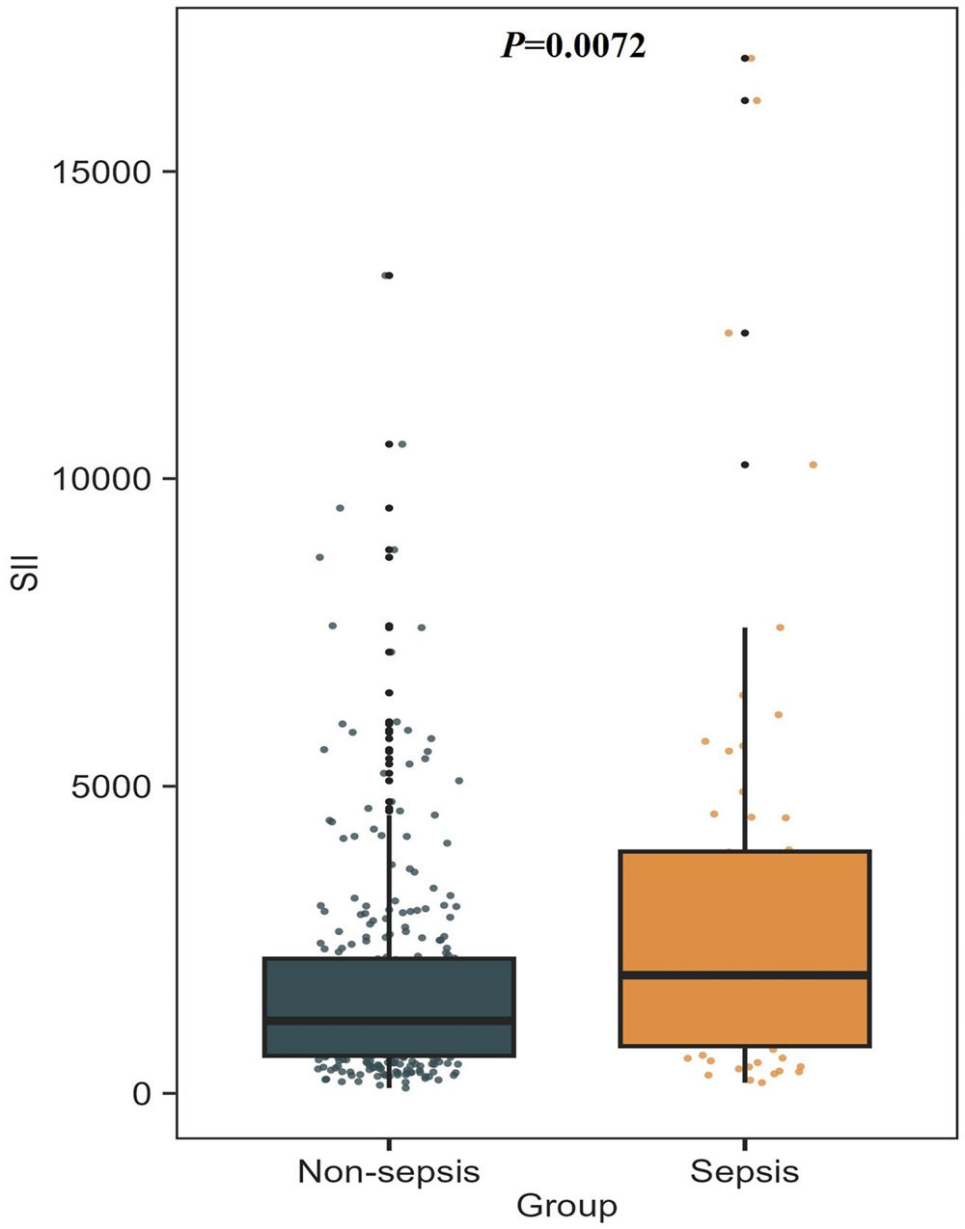
The box plot based on the systemic immune inflammation index (SII) values. The Wilcoxon test was used to compare the difference in the SII values between the nonsepsis group and sepsis group (*p* = .0072).

**Figure 3:**
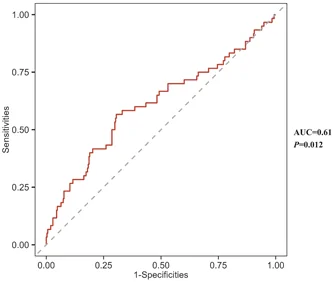
Receiver operating curve of the systemic inflammatory index. The systemic immune inflammation index for postoperative sepsis had an AUC of 0.610 (*p* = .012).

**Figure 4:**
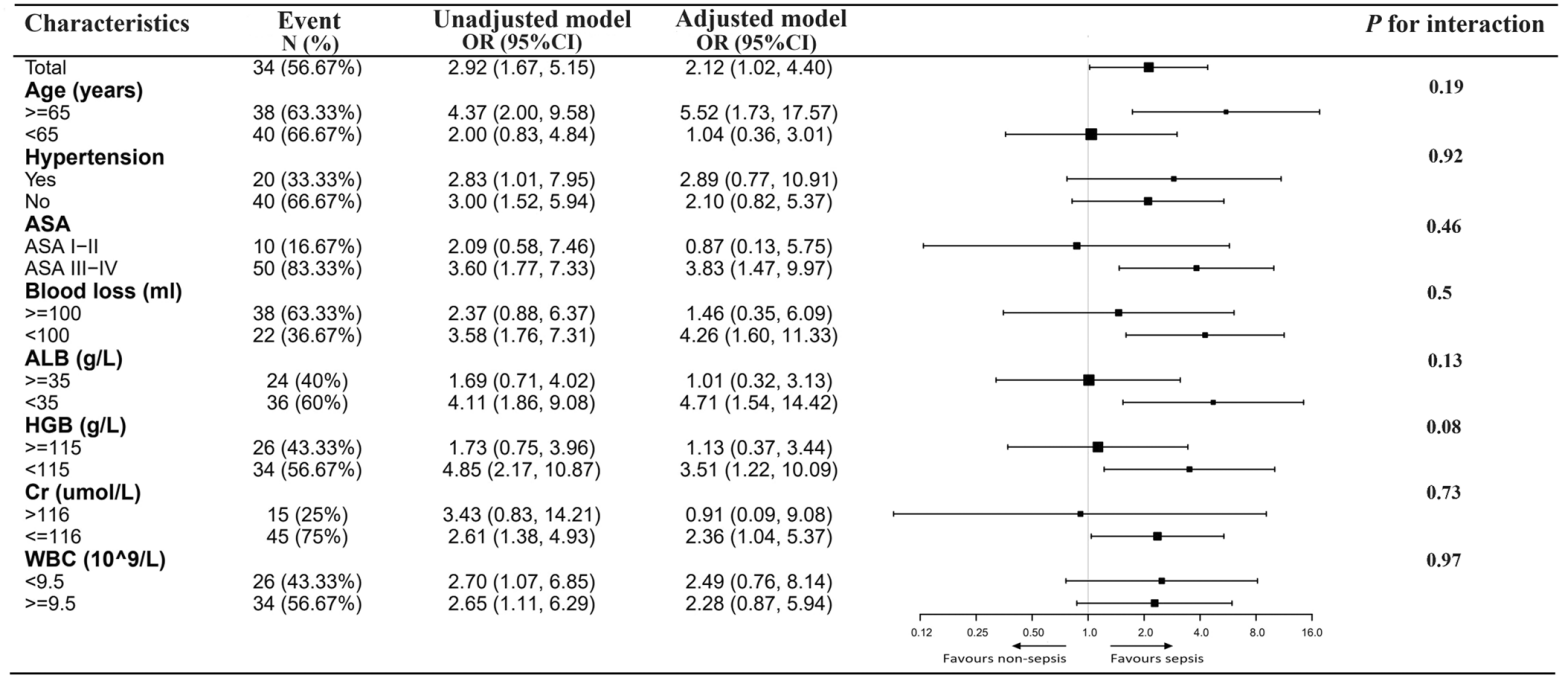
Forest plot for subgroup analysis. Unadjusted model: using the univariate logistic model; adjusted model: using multivariate logistic regression including the predefined confounders of age, duration of surgery, and white blood cell count; P for interaction: result of the interaction analysis. ASA, American Society of Anesthesiologists physical status classification system; ALB, albumin; CR, creatinine; HGB, hemoglobin; WBC, white blood cell count.

The box plot shows that the median, 75th percentiles, and maximum values of SII in the sepsis group are higher than those in the nonsepsis group (1923.92 vs. 1180.98, 5146.31 vs. 2771.65, 16842.43 vs. 13306.53, *p* = .0072, Figure 2). The ROC analysis determined 1792.19 as the ideal cut‐off value for the SII. Following Figure 3, the SII for postoperative sepsis had a cut‐off value of 1792.19, AUC of 0.610, a sensitivity of 56.67%, and a specificity of 69.45%. To explore the relationship between the SII and postoperative outcomes, we found that those with a preoperative SII ＞1792.19 were associated with a higher incidence of postoperative sepsis (26.15% vs. 10.79%, *p* < .05; Table 1), longer time of drainage tube retention, longer postoperative hospitalization days and a longer ICU length of stay (all *p* < .05; Table 1). Post hoc power analysis demonstrated a study power of 0.96, which shows study was sufficiently powered. The correlation analysis revealed that individuals with a preoperative SII ＞1792.19 were at a higher risk of developing postoperative sepsis (odds ratio [OR] = 2.92, 95% confidence interval [CI]: [1.67–5.15], *p* < .05). We adjusted the results taking into account confounding factors such as age, WBC, surgical duration, cause of obstruction, and intestinal state. After multivariable adjustment, further correlation analysis revealed that individuals with a preoperative SII ＞1792.19 were at a higher risk of developing postoperative sepsis (adjusted odds ratio [aOR] = 2.12, 95% CI: [1.02–4.40], *p* < .05; Figure 4). Furthermore, the analysis of interaction showed no correlation between the preoperative SII and postoperative sepsis regarding age, hypertension, ASA classification, blood loss, ALB, HGB, CR, and leukocyte (all interactions *p* > .05). In subgroup analysis, all statistically significant subgroups showed that SII was a risk factor for postoperative sepsis, such as age ≥65 years old (OR = 5.52, 95% CI: [1.73–17.57], *p* < .001), ASA III–IV (OR = 3.83, 95% CI: [1.47–9.97], *p* < .001), blood loss <100 mL (OR = 4.26, 95% CIL [1.60–11.33], *p* < .001), ALB ＜35 g/L (OR = 4.71, 95% CI: [1.54–14.42], *p* < .001), HGB ＜115 g/L (OR = 3.51, 95% CI: [1.22–10.09], *p* = .001), Cr ＜116 μmol/L (OR = 2.36, 95% CI: [1.04–5.37], *p* = .006).

### 3.3 Sensitivity analyses

**Figure 5:**
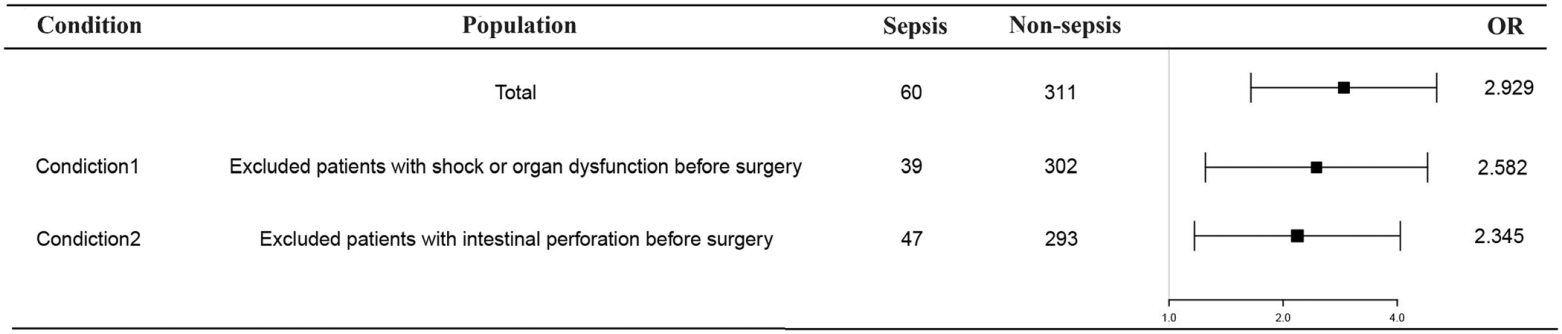
Sensitivity analysis by adjusting for specific confounders of specified properties. OR, odds ratio.

To consider the observed confounders, we conducted sensitivity analyses (Figure 5). First, even after excluding patients who experienced shock or organ dysfunction before surgery, the result remained significant. Sepsis was more common for an SII ＞1792.19 compared to an SII ≤ 1792.19 (OR = 2.582, 95% CI: [1.314–5.070], *p* < .05). Furthermore, we reanalyzed the data after eliminating patients who had intestinal perforation before surgery; the risk of sepsis was somewhat increased (OR = 3.026, 95% CI: [1.615–5.669], *p* < .05).

## 4 DISCUSSION

In this retrospective observational cohort study, intestinal obstruction patients with a preoperative SII ＞1792.19 had a significantly increased risk of developing postoperative sepsis, with an aOR of 2.12 (95% CI: 1.02–4.40). Additionally, the association between SII and postoperative sepsis in patients with intestinal obstruction was not impacted by other confounding variables, based on subgroup analysis and interaction validation results. Sensitivity analyses were also conducted to rule out the possibility of reverse causality. However, the conclusion was not different from those of the entire cohort.

The SII combines NLR with PLT to represent the balance between the inflammation, immune response, and thrombotic pathways. Large quantities of pro‐inflammatory cytokines are released during the complicated pathophysiological process of sepsis, which causes inflammatory reactions throughout the body and promotes its progression.^23^ Additionally, the various anti‐inflammatory cytokines released into the circulation may induce immunosuppression, subsequently leading to many lymphocytes undergoing apoptosis.^24^ The NEUT increases sharply during sepsis because neutrophils react rapidly to microbial infection. These neutrophils then migrate to the affected area, but they have also transitioned from powerful antibacterial protectants to potentially dangerous mediums that can cause tissue damage and organ dysfunction.^25^ More than 40 years ago, thrombocytopenia and sepsis were first shown to be related.^26^ In patients with sepsis, thrombocytopenia is a potent prognostic indicator that is considered to arise from platelet activation and consumption.^27^, ^28^ Because the NEUT and PLT in the numerator and lymphocytes are included in the denominator, a higher neutrophil or PLT or a lower LYMPH will result in a higher SII value.

The SII was associated with the prognosis of patients with hepatocellular carcinoma after curative resection in the beginning.^29^ In general, SII has been found to have predictive value for the prognosis of various tumors.^30^, ^31^, ^32^ Meanwhile, most studies have shown that a higher SII is associated with the occurrence and poor prognosis of sepsis.^21^, ^33^ However, a retrospective study showed that not only is a higher SII associated with the death of critically ill patients with sepsis, but a lower SII also led to an increased risk of short‐term death.^22^ Low SII levels theoretically signal that the body may be suffering from significant inflammation and immune suppression disorder. However, intestinal obstruction patients who are about to undergo surgical treatment rarely experience these conditions before surgery. The SII is also a potential biomarker for other diseases. Man Xu et al. reported in a retrospective cohort study of 13,929 middle‐aged and elderly people that SII can be used as a useful indicator to clarify the interaction between thrombocytosis and immune inflammation in the occurrence and development of cerebral vascular disease in middle‐aged and elderly people.^34^ Other studies also reported the potential role of SII in cardiocerebral vascular disease.^35^, ^36^, ^37^ A higher SII has also been associated with an increased risk of POCD,^38^ rheumatoid arthritis,^39^ and postoperative pulmonary complications.^40^

The PLT, neutrophil, and LYMPHs could all be examined routinely and easily in patients undergoing selective and emergency procedures, making the marker available to doctors in our daily practice. Additionally, SII can easily be integrated into hospital information systems and EHRs, allowing it to be used as a clinical decision‐making support tool for patients with postoperative intestinal obstruction. Preoperative SII may present anesthesiologists with diagnostic and prognostic evidence to identify sepsis in patients undergoing intestinal obstruction surgery, allowing them to better avoid and treat it. It should be clarified that preoperative SII evaluation only begins when patients have determined the need for surgery. SII is not a tool for determining the timing of surgery, it is used to quickly identify high‐risk patients who are prone to sepsis after intestinal obstruction surgery. Preoperative use of antibiotics and anti‐inflammatory drug can improve the systemic inflammatory response status of patients and reduce SII levels, which helps to reduce the risk of postoperative sepsis in such patients. Intraoperative fluid resuscitation for high‐risk patients can also reduce the risk of postoperative sepsis. This will also help with research into how the immune–inflammatory response can lead to sepsis, which will provide a better understanding of the pathophysiology of a range of dysfunctions produced by sepsis. However, it should be clarified that this study only confirmed that SII ＞1792.19 may be a potential risk factor for postoperative sepsis in patients with intestinal obstruction, but failed to confirm that SII can predict the incidence of postoperative sepsis, despite its AUC of 0.610 and sensitivity of 56.7% indicates moderate predictive or diagnostic performance. Due to the limitations of retrospective study designs, we could not determine the precise impact of anti‐inflammatory therapy on patients with the same condition. Further prospective clinical studies should be conducted to determine whether preoperative anti‐inflammatory medication might improve patients' results and when and how to manage them.

Our study has some limitations. First, it was a single‐center study with limited sample size, resulting in a small number of cases in subgroup analysis and insignificant results. This may have led to failure to identify populations in which it may be more suitable to use the SII to identify and predict postoperative sepsis in patients with intestinal obstruction. Second, only the variables present in the EHR system were used in the analysis. Therefore, it is impossible to entirely rule out additional potentially significant confounding factors. Third, although this study first discovered a correlation between preoperative SII and postoperative sepsis in patients with intestinal obstruction surgery, its AUC and sensitivity were not outstanding enough. Whether SII has better guidance value and predictive ability in a certain subgroup population (combined with preoperative diseases, preoperative intervention measures, etc.) still needs to include more cases and conduct more research. Fourth, further analysis of any correlation between SII and SOFA scores is valuable for the conclusion that SII can identify high‐risk patients with sepsis. This requires further analysis in the future. Fifth, more studies are needed in the future to confirm the prognostic value of the components, as it will enhance the importance and potential use of SII as a more valuable clinical tool than its sum. Sixth, our study did not compare with other inflammatory markers, so we cannot determine if there is a better correlation between other inflammatory markers and postoperative sepsis in such patients. Finally, to investigate the effect of preoperative anti‐inflammatory medication on postoperative sepsis, the findings still require further verification through prospective randomized controlled trials.

## 5 CONCLUSIONS

In sum, a preoperative SII ＞1792.19 was a potential risk factor for postoperative sepsis in patients with intestinal obstruction. Based on the SII value, we can identify high‐risk patients who develop sepsis after intestinal obstruction surgery as early as possible.

## AUTHOR CONTRIBUTIONS

**Jirong Yang**: Conceptualization; formal analysis; investigation; methodology; writing—original draft preparation. **Taojia Ran**: Investigation; methodology. **Xiaoyu Lin**: Investigation; validation. **Jinyan Xu**: Investigation. **Shaoli Zhou**: Project administration; resources; supervision. **Chaojin Chen**: Conceptualization; data curation; project administration; funding acquisition; supervision; writing—review and editing. **Pinjie Huang**: Conceptualization; data curation; project administration; resources; supervision; writing—review and editing.

## CONFLICT OF INTEREST STATEMENT

The authors declare no conflict of interest.

## ETHICS STATEMENT

Ethical approval of this study was obtained from the Research Ethics Committee at the Third Affiliated Hospital of Sun Yat‐sen University (NO. [2022] 02‐004‐02). This study was exempted from having to obtain informed consent because the patient identities would not be recognized, and perioperative data were obtained from electrical health records (EHR).

## Data Availability

Under existing ethical approvals, the availability of these data is limited and therefore cannot be publicly obtained. However, upon reasonable request and with the permission of the author and the Third Affiliated Hospital of Sun Yat‐sen University, data may be provided.

## References

[1] Singer M , Deutschman CS , Seymour CW , et al. The third international consensus definitions for sepsis and septic shock (Sepsis‐3). JAMA. 2016;315:801‐810. 10.1001/jama.2016.0287 26903338 PMC4968574

[2] Fleischmann C , Scherag A , Adhikari NKJ , et al. Assessment of global incidence and mortality of hospital‐treated sepsis. current estimates and limitations. Am J Respir Crit Care Med. 2016;193:259‐272. 10.1164/rccm.201504-0781OC 26414292

[3] Vincent JL , Marshall JC , Ñamendys‐Silva SA , et al. Assessment of the worldwide burden of critical illness: the intensive care over nations (ICON) audit. Lancet Respir Med. 2014;2:380‐386. 10.1016/S2213-2600(14)70061-X 24740011

[4] Rhodes A , Evans LE , Alhazzani W , et al. Surviving sepsis campaign: international guidelines for management of sepsis and septic shock: 2016. Intensive Care Med. 2017;43:304‐377. 10.1007/s00134-017-4683-6 28101605

[5] Vincent JL . The clinical challenge of sepsis identification and monitoring. PLoS Med. 2016;13:e1002022. 10.1371/journal.pmed.1002022 27187803 PMC4871479

[6] de Grooth HJ , Postema J , Loer SA , Parienti JJ , Oudemans‐van Straaten HM , Girbes AR . Unexplained mortality differences between septic shock trials: a systematic analysis of population characteristics and control‐group mortality rates. Intensive Care Med. 2018;44:311‐322. 10.1007/s00134-018-5134-8 29546535 PMC5861172

[7] Peltan ID , Brown SM , Bledsoe JR , et al. ED door‐to‐antibiotic time and long‐term mortality in sepsis. Chest. 2019;155:938‐946. 10.1016/j.chest.2019.02.008 30779916 PMC6533450

[8] Rüddel H , Thomas‐Rüddel DO , Reinhart K , et al. Adverse effects of delayed antimicrobial treatment and surgical source control in adults with sepsis: results of a planned secondary analysis of a cluster‐randomized controlled trial. Crit Care. 2022;26:51. 10.1186/s13054-022-03901-9 35227308 PMC8883454

[9] Van Leeuwen PA , Boermeester MA , Houdijk AP , et al. Clinical significance of translocation. Gut. 1994;35:S28‐S34. 10.1136/gut.35.1_suppl.s28 8125386 PMC1378143

[10] Proctor DW , Goodall R , Borsky K , et al. Trends in the mortality, incidence, and disability‐adjusted life‐years of intestinal obstruction and paralytic ileus: observational study of the Global Burden of Disease database. Br J Surg. 2023;110(12):1650. 10.1093/bjs/znad232 37531553

[11] Jackson P , Vigiola Cruz M . Intestinal obstruction: evaluation and management. Am Fam Physician. 2018;98:362‐367. 30215917

[12] Van Leeuwen PA , Boermeester MA , Houdijk AP , et al. Clinical significance of translocation. Gut. 1994;35:S28‐S34. 10.1136/gut.35.1_suppl.s28 8125386 PMC1378143

[13] Hegde S , Lin Y‐M , Golovko G , et al. Microbiota dysbiosis and its pathophysiological significance in bowel obstruction. Sci Rep. 2018;8:13044. 10.1038/s41598-018-31033-0 30177854 PMC6120911

[14] Dragoescu AN , Padureanu V , Stanculescu AD , et al. Neutrophil to lymphocyte ratio (NLR)—a useful tool for the prognosis of sepsis in the ICU. Biomedicines. 2021;10(1):75. 10.3390/biomedicines10010075 35052755 PMC8772781

[15] Wacker C , Prkno A , Brunkhorst FM , Schlattmann P . Procalcitonin as a diagnostic marker for sepsis: a systematic review and meta‐analysis. Lancet Infect Dis. 2013;13:426‐435. 10.1016/S1473-3099(12)70323-7 23375419

[16] Yu Y , Wu W , Dong Y , Li J . C‐reactive protein‐to‐albumin ratio predicts sepsis and prognosis in patients with severe burn injury. Mediators Inflamm. 2021;2021:6621101. 10.1155/2021/6621101 33833617 PMC8016580

[17] Huang Y , Gao Y , Wu Y , Lin H . Prognostic value of systemic immune‐inflammation index in patients with urologic cancers: a meta‐analysis. Cancer Cell Int. 2020;20:499. 10.1186/s12935-020-01590-4 33061851 PMC7552553

[18] Li J , Cao D , Huang Y , et al. The prognostic and clinicopathological significance of systemic immune‐inflammation index in bladder cancer. Front Immunol. 2022;13:865643. 10.3389/fimmu.2022.865643 35572533 PMC9097688

[19] Jomrich G , Paireder M , Kristo I , et al. High systemic immune‐inflammation index is an adverse prognostic factor for patients with gastroesophageal adenocarcinoma. Ann Surg. 2021;273:532‐541. 10.1097/SLA.0000000000003370 31425286

[20] Dong M , Shi Y , Yang J , et al. Prognostic and clinicopathological significance of systemic immune‐inflammation index in colorectal cancer: a meta‐analysis. Ther Adv Med Oncol. 2020;12:1758835920937425. 10.1177/1758835920937425 32699557 PMC7357045

[21] Mangalesh S , Dudani S , Malik A . The systemic immune‐inflammation index in predicting sepsis mortality. Postgrad Med. 2023;135:345‐351. 10.1080/00325481.2022.2140535 36287784

[22] Jiang D , Bian T , Shen Y , Huang Z . Association between admission systemic immune‐inflammation index and mortality in critically ill patients with sepsis: a retrospective cohort study based on MIMIC‐IV database. Clin Exp Med. 2023;23(7):3641‐3650. 10.1007/s10238-023-01029-w 36930382 PMC10022570

[23] Font MD , Thyagarajan B , Khanna AK . Sepsis and septic shock—basics of diagnosis, pathophysiology and clinical decision making. Med Clin North Am. 2020;104:573‐585. 10.1016/j.mcna.2020.02.011 32505253

[24] Torres LK , Pickkers P , van der Poll T . Sepsis‐induced immunosuppression. Annu Rev Physiol. 2022;84:157‐181. 10.1146/annurev-physiol-061121-040214 34705481

[25] Moriyama K , Nishida O . Targeting cytokines, pathogen‐associated molecular patterns, and damage‐associated molecular patterns in sepsis via blood purification. Int J Mol Sci. 2021;22(16):8882. 10.3390/ijms22168882 34445610 PMC8396222

[26] Bone RC , Francis PB , Pierce AK . Intravascular coagulation associated with the adult respiratory distress syndrome. Am J Med. 1976;61:585‐589. 10.1016/0002-9343(76)90135-2 984062

[27] Giustozzi M , Ehrlinder H , Bongiovanni D , et al. Coagulopathy and sepsis: pathophysiology, clinical manifestations and treatment. Blood Rev. 2021;50:100864. 10.1016/j.blre.2021.100864 34217531

[28] Vardon‐Bounes F , Ruiz S , Gratacap MP , Garcia C , Payrastre B , Minville V . Platelets are critical key players in sepsis. Int J Mol Sci. 2019;20(14):3494. 10.3390/ijms20143494 31315248 PMC6679237

[29] Hu B , Yang XR , Xu Y , et al. Systemic immune‐inflammation index predicts prognosis of patients after curative resection for hepatocellular carcinoma. Clin Cancer Res. 2014;20:6212‐6222. 10.1158/1078-0432.CCR-14-0442 25271081

[30] Meng L , Yang Y , Hu X , Zhang R , Li X . Prognostic value of the pretreatment systemic immune‐inflammation index in patients with prostate cancer: a systematic review and meta‐analysis. J Transl Med. 2023;21:79. 10.1186/s12967-023-03924-y 36739407 PMC9898902

[31] Nøst TH , Alcala K , Urbarova I , et al. Systemic inflammation markers and cancer incidence in the UK Biobank. Eur J Epidemiol. 2021;36:841‐848. 10.1007/s10654-021-00752-6 34036468 PMC8416852

[32] Zhang K , Hua YQ , Wang D , et al. Systemic immune‐inflammation index predicts prognosis of patients with advanced pancreatic cancer. J Transl Med. 2019;17:30. 10.1186/s12967-019-1782-x 30658662 PMC6339361

[33] Ma K , Zhang Y , Hao J , Zhao J , Qi Y , Liu C . Correlation analysis of systemic immune inflammatory index, serum IL‐35 and HMGB‐1 with the severity and prognosis of sepsis. Pak J Med Sci. 2023;39:497‐501. 10.12669/pjms.39.2.6651 36950402 PMC10025737

[34] Xu M , Chen R , Liu L , et al. Systemic immune‐inflammation index and incident cardiovascular diseases among middle‐aged and elderly Chinese adults: the Dongfeng‐Tongji cohort study. Atherosclerosis. 2021;323:20‐29. 10.1016/j.atherosclerosis.2021.02.012 33773161

[35] Orhan AL , Şaylık F , Çiçek V , Akbulut T , Selçuk M , Çınar T . Evaluating the systemic immune‐inflammation index for in‐hospital and long‐term mortality in elderly non‐ST‐elevation myocardial infarction patients. Aging Clin Exp Res. 2022;34:1687‐1695. 10.1007/s40520-022-02103-1 35275375

[36] Peng F , Xia J , Niu H , et al. Systemic immune‐inflammation index is associated with aneurysmal wall enhancement in unruptured intracranial fusiform aneurysms. Front Immunol. 2023;14:1106459. 10.3389/fimmu.2023.1106459 36776878 PMC9911448

[37] Su S , Liu J , Chen L , et al. Systemic immune‐inflammation index predicted the clinical outcome in patients with type‐B aortic dissection undergoing thoracic endovascular repair. Eur J Clin Invest. 2022;52:e13692. 10.1111/eci.13692 34695253

[38] Song Y , Luo Y , Zhang F , et al. Systemic immune‐inflammation index predicts postoperative delirium in elderly patients after surgery: a retrospective cohort study. BMC Geriatr. 2022;22:730. 10.1186/s12877-022-03418-4 36064357 PMC9446812

[39] Liu B , Wang J , Li Y , Li K , Zhang Q . The association between systemic immune‐inflammation index and rheumatoid arthritis: evidence from NHANES 1999‐2018. Arthritis Res Ther. 2023;25:34. 10.1186/s13075-023-03018-6 36871051 PMC9985219

[40] Mao X , Zhang W , Wang Q , Ni Y , Niu Y , Jiang L . Assessment of systemic immune‐inflammation index in predicting postoperative pulmonary complications in patients undergoing lung cancer resection. Surgery. 2022;172:365‐370. 10.1016/j.surg.2021.12.023 35101329

